# Syphilitic Affections of the Mouth

**Published:** 1898-03

**Authors:** Henry H. Burchard


### SYPHILITIC AFFECTIONS OF THE MOUTH.[1]

BY HENRY H. BURCHARD, M.D., D.D.S.

The recognition of syphilitic lesions about the mouth is of vital importance to the dental operator, first, because by their recognition he may take steps to prevent the carriage of infection to innocent patients, and, secondly, that he may avoid inoculation of himself by the poison.

Syphilis is associated in the minds of many with the lower classes of persons who are confirmed debauchees; while it is undoubtedly true that its prevalence is most marked in this class of persons, it appears, and with horrible frequence, in individuals who would be little suspected of having such infection. The operator is to be guided in his opinions and precautions in this matter not by the social status of the patient but by the nature of the morbid conditions existing.

The disease syphilis is usually divided into three stages: primary, secondary, and tertiary. To these might be added a fourth stage, —viz., in patients who have been discharged as cured. Mild manifestations of disorders, particularly of the skin and mucous membrane, make their appearance from time to time, and disappear promptly upon the administration of iodides.

The first stage of syphilis, primary syphilis, consists in the formation of the primary sore or chancre and the involvement of the nearest lymphatic glands.

Secondary syphilis appears after the first, and is attended by fever, inflammations of the skin with eruptions, inflammation and superficial ulcerations of mucous structures.

In tertiary syphilis destructive inflammation of the skin and mucous membranes and of connective tissues occur, together with the formation of specific tumors, gummata. Some difference of opinion exists among syphilographers as to the relative infective power of the secretions from the several lesions of syphilis; all are agreed, however, that the secretions from the secondary lesions observed in and about the mouth are highly infective. It is the part of prudence to regard lesions of all stages as infective.

All three stages of syphilis may be seen in the human mouth.

---

[1] Excerpt from chapter on Infections in Dr. Burchard's new text-book of "Dental Pathology and Therapeutics." Lea Brothers & Co., 1898. (In press.) Read before the Academy of Stomatology, December 28, 1897.

It is to be remembered that if the mucous membrane of the mouth be infected from a mucous patch, which is a secondary lesion, the acquired disease appears not as a mucous patch but as a chancre. It is from mucous patches that infection is most to be feared in dental practice.

PRIMARY SYPHILIS OF THE MOUTH.

The primary lesion of syphilis, chancre, is found in the mouth in consequence of direct infection, carried from a syphilitic. The infection may occur from contact of the mucous surface of the mouth with a syphilitic lesion upon another person. It has been transmitted by kissing. It may occur from using a glass or cup previously used by a syphilitic, by smoking cigars or cigarettes which have been made by syphilitic cigar-makers who have applied the tongue to the tobacco in attaching the wrapper.

Any of the articles named or the contact of any article which has been in contact with a syphilitic lesion, if brought in contact with an abraded mucous surface, is very likely to cause infection. The infection may be transferred from patient to operator if the fingers have any abraded surface, or if the surface is broken accidentally by an instrument. Infection may be transmitted from one patient to another by any instrument, appliance, or article used by the syphilitic being afterwards used by an innocent person. Drinking-glasses, mouth-mirrors, exploring instruments, rubber dam, rubber-dam clamps, lancets, forceps, or any other instruments, may be the medium of communication. During and since the time of Hunter, the use of teeth from syphilitic patients in plantation operations has been a clearly recognized medium of communication.

"The primary lesion of syphilis never makes its appearance before ten days after infection; the maximum period is about ninety days, the average is twenty-one days." (Gross.)

It usually appears as a single elevated papule; in cases of dental infection frequently about the lips; the papule loses its epithelial coating after some days. The hardening surrounding the papular mass increases until the papule, which is now raw and in a process of ulceration, appears surrounded by a ring of cartilaginous hardness. This induration is the one distinguishing feature of the chancre, which is not painful. In about a week after the appearance of the primary sore swelling of the submaxillary lymphatic glands is observed. "In case the chancre appears upon the tongue, the subhyoid lymphatic glands are swollen" (Park). Unless pyogenic infection has occurred, the lymphatic involvement is not inflammatory, there being no pain in the glands.

In from three to four weeks the sore disappears; in some cases leaving no sign of its site, in others some induration may persist.

The diagnosis of this condition is the important consideration so far as the dental practitioner is concerned, its constitutional treatment being the office of the general surgeon.

The elevation of the sore, its induration, and, if obtainable, the date of inoculation are diagnostic data. The sore is single, and there is hard, nodular, painless swelling of the neighboring lymphatics. A single ulcer of ulcerative stomatitis may in some degree simulate the appearance of chancre, for it may exhibit slight induration. Their irregular form, situation, painfulness, and usual absence of lymphatic involvement, together with their prompt disappearance after sterilizing the mouth and cauterizing of the ulcer, will differentiate the two sores.

It is a wise precaution, however, to view all sores about the mouth as possibly infective; all errors of diagnosis in this direction will be more than compensated for by the assurance of non-transferrence of infection.

### SECONDARY SYPHILIS OF THE MOUTH.

The secondary manifestations of syphilis are observed in and about the mouth, no matter where the location of the primary lesion may have been; they are the result of a general, not a local, infection.

Secondary affections of the mucous tissues appear in from four to twelve weeks after the appearance of the primary lesion. Sore throat due to inflammation of the mucous membrane of the pharynx and parts about is almost constant, together with syphilitic hoarseness due to the extension of the affection to the mucous membrane of the larynx. Symmetrical and usually painless, shallow ulcers of the tonsils are characteristic. The appearance of copper-colored areas upon some portion of the mucous membrane, on the tonsil, pharynx, soft palate, lips, or bucco-labial surface, precedes the loss of epithelium over such surfaces, which soon occurs, forming the most virulently contagious lesion of syphilis, the mucous patch.

These become covered with a grayish-white pasty coating resembling the ulcerations of non-specific stomatitis; so close is the resemblance that a differentiation can only be made at times by additional evidences of secondary syphilis. Single patches may coalesce, forming large, irregular areas covered by a grayish-white pellicle. These patches are rarely painful. Ulcerations having ragged, irregular outlines may appear at the sites of the original patches or in other situations which exhibit a tendency to spread. The diagnosis of the condition is determined by a discovery of other lesions of secondary syphilis, skin-eruptions, falling out of the hair (alopecia), and by the areas of copper-colored eruption upon the mucous membrane of the pharynx and soft palate. Hugenschmidt has found the frequent nocturnal occurrence of indefinitely located dental pains spreading to the palatal region in the absence of local lesions.

### TERTIARY SYPHILIS OF THE MOUTH.

The syphilides of the secondary stage arise in and are confined to the mucous and submucous structures of the mouth; those of the tertiary stage arise in the deep connective tissues, and are frequently associated with periosteum.

Tertiary lesions as seen by the dentists are usually in the form of ulcers, of first the soft or hard palate of the tongue or lips. In the earlier stages hard, nodular formations may be noted as antecedents to the ulcerations. Chronic periostitis of the palatal process may occur, leading to the formation of localized thickenings. In other cases localized swellings in the soft palate may occur; the overlying mucous membrane breaks, establishing an ulcer, which may perforate the soft palate, destroy a portion of the palatal process, or form large ulcers on the tongue. These lesions appear in from two to five years after the secondary manifestations. Although there is much doubt as to the infectiveness of these tertiary lesions, precautions as to sterilization should be taken as with the primary and secondary lesions.

A defined ragged ulcer occupying the hard or soft palate, which has persisted for a long time, is always viewed with suspicion, and a search is made for other evidences of syphilis. These ulcerations appearing upon the side of the tongue may closely simulate epithelioma of that organ; the confusion is increased if in consequence of the presence of jagged teeth a continuous irritation is excited. Moreover, leukoplakia of the cheeks frequently accompanies tertiary syphilis. An absolute diagnosis is only made out in some cases by noting the disappearance of the local lesion upon the administration of iodides, the specific treatment of tertiary syphilis.

In any of these conditions, particularly those named under the head of secondary affections, extraordinary precautions should be taken to prevent transmission of infection. Every instrument or article brought in contact with the mouth of the patient should be set aside for special sterilization. Napkins used upon such patients should be thrown away; the same applies, of course, to rubber dam and saliva tube ejectors. All other articles should be immersed in an antiseptic solution, say a five-per cent. lysol solution, and be afterwards boiled for fifteen minutes in a two-per-cent. solution of sodium carbonate, not bicarbonate.

Forceps, rubber-dam clamps, and mouth-mirrors need special attention. Should the fingers of the operator have any abrasions upon them, it is the part of prudence not to insert them in the syphilitic mouth, certainly never without first covering the abrasions with collodion or an impenetrable varnish.

The above excerpt is as much material relative to this subject as the plan of the text-book from which it is taken admits. This might be expanded to any length, however. The principal object in presenting this matter, with the illustrations, is to point out the more usual oral manifestations of syphilis, their relative infectiousness, and their diagnosis.

The picture of the chancre represents an outlined sore upon the lower lip; similar sores may be seen upon the gums, the tongue, the pharynx, and other parts. If on the tongue, they may be very large instead of small, as here represented; the mechanical irritation of bruising against irregular teeth, or their sharp edges, may cause very angry-looking and extensive sores.

This applies also to the secondary sore which is depicted under similar conditions, and particularly in the mouths of confirmed smokers, where secondary lesions are most extensive. Under these conditions the lesions of secondary syphilis may acquire the appearance of tertiary lesions. This is of decided importance, because it is the lesions of secondary syphilis which are the highly infective ones, even more, the saliva is probably infective, whereas it is doubtful whether tertiary lesions are infective.

The gumma in the palate is represented in the submucous tissue prior to discharge and ulceration. The phagedenic nature of these tertiary sores is shown in the lesion of the lower lip where the deep muscles of the lip are invaded.

I show you also representations of Hutchinson's teeth, diagnostic signs of hereditary syphilis. You will observe that the malformation is confined to the upper central incisors; this is a general but by no means universal truth, for the malformation may involve the other teeth also; this, however, is unusual. Williams has shown that the dentine of such teeth is very badly organized, as is also the enamel; but the formative organ of the latter tissue is disordered in function and form; not only is the enamel built with great irregularity as to its elements, but the functional activity of the ameloblasts ceases too soon. The teeth formed during the eruptive fevers are shown in contrast; it will be seen in these, that while the teeth have almost their normal forms, enamel-building has been checked at an early period, affecting all of the developing tooth, but when the nutritive equilibrium is re-established enamel deposit proceeds normally.

